# Factors Affecting Exercise Tolerance in Patients Who Underwent Video-Assisted Thoracoscopic Surgery for Lung Cancer

**DOI:** 10.7759/cureus.85956

**Published:** 2025-06-13

**Authors:** Yoshiteru Akezaki, Makiko Hamada, Ritsuko Tominaga, Masaki Okamoto, Hideaki Kurokawa, Masato Kikuuchi, Tsuyoshi Ueno, Motohiro Yamashita, Eiji Nakata, Shinsuke Sugihara

**Affiliations:** 1 Division of Physical Therapy, Kochi Professional University of Rehabilitation, Kochi, JPN; 2 Department of Rehabilitation Medicine, National Hospital Organization Ehime Medical Center, Ehime, JPN; 3 Department of Rehabilitation Medicine, National Hospital Organization Shikoku Cancer Center, Ehime, JPN; 4 Department of Rehabilitation Medicine, National Hospital Organization Kochi Hospital, Kochi, JPN; 5 Department of Thoracic Surgery, National Hospital Organization Shikoku Cancer Center, Ehime, JPN; 6 Department of Orthopaedic Surgery, Okayama University Hospital, Okayama, JPN

**Keywords:** exercise tolerance, handgrip strength, lung cancer, physical activity, rehabilitation

## Abstract

Background

Video-assisted thoracoscopic surgery (VATS) is increasingly being performed instead of open thoracotomy for early-stage lung cancer because of the minimally invasive nature of VATS. In this study, we aimed to identify the factors related to exercise tolerance in patients with lung cancer who underwent VATS.

Methods

in this study, we included 73 consecutive patients who underwent video-assisted lung lobectomy for lung cancer at Shikoku Cancer Center, Matsuyama, Japan. Clinical parameters like the presence or absence of *c*hronic obstructive pulmonary disease (COPD), operative time, intraoperative bleeding, duration of postoperative drain placement, skeletal muscle mass index, muscle strength, physical activity, and exercise tolerance were assessed. Muscle strength was measured using handgrip strength. Exercise tolerance was measured according to the 6-minute walk distance.

Results

After surgery, handgrip strength and six-minute walk distance were significantly lower than those observed during the preoperative evaluation of the patients (p < 0.05). Stepwise multiple regression analyses showed that higher postoperative handgrip strength, males, and higher preoperative physical activity had a significant positive effect on postoperative six-minute walk distance (p < 0.05, R^2^ = 0.348).

Conclusions

Improving postoperative exercise tolerance also requires interventions to increase physical activity before surgery and postoperative interventions that also focus on muscle strength.

## Introduction

Lung cancer is the most frequently diagnosed cancer and the leading cause of cancer-related deaths worldwide [1]. The major components of lung cancer treatment are chemotherapy, radiotherapy, and surgery [2], with lung lobectomy generally considered the optimal treatment for early-stage lung cancer [3]. Video-assisted thoracoscopic surgery (VATS) is increasingly being performed instead of open thoracotomy for early-stage non-small cell lung cancer (NSCLC) because of the minimally invasive nature [4].

After lung resection, reduced functional capacity is common postoperatively due to systemic inflammation and surgical stress [3], and patients with lung cancer show significantly reduced exercise tolerance within 14 days of surgery [5,6]. These patients might not regain their baseline physical performance levels for several months [7]. 

The six-minute walk test (6MWT) is an inexpensive measure of physical function and an important indicator of exercise tolerance, including cardiovascular, pulmonary, and neuromusculoskeletal performance [8]. Distance covered in the 6MWT is decreased because of several diseases such as heart failure, chronic obstructive pulmonary disease (COPD), and stroke [9-11]. In patients with congestive heart failure and COPD, the six-minute walk distance (6MWD) shows a correlation with the cardiopulmonary capacity, which is determined by measuring the maximum oxygen uptake that occurs during a bicycle exercise or a treadmill test [12,13]. The 6MWT has also been shown to predict postoperative complications and survival in patients undergoing pulmonary surgery [14]. However, our search on PubMed for the keywords “VATS” and “6-minute walk” or “exercise tolerance” did not reveal any studies examining factors related to endurance in patients who underwent VATS for lung cancer. Rehabilitation of postoperative lung cancer patients is performed not only for patients who have undergone open thoracotomy but also for those who have undergone VATS. Identification of the factors related to 6MWD will help medical staff in planning rehabilitation strategies for these patients.

In this study, we aimed to identify the factors related to exercise tolerance in patients who underwent VATS for lung cancer.

## Materials and methods

Study design

This is a retrospective, observational study of the factors related to exercise tolerance.

Patients

**Figure 1:**
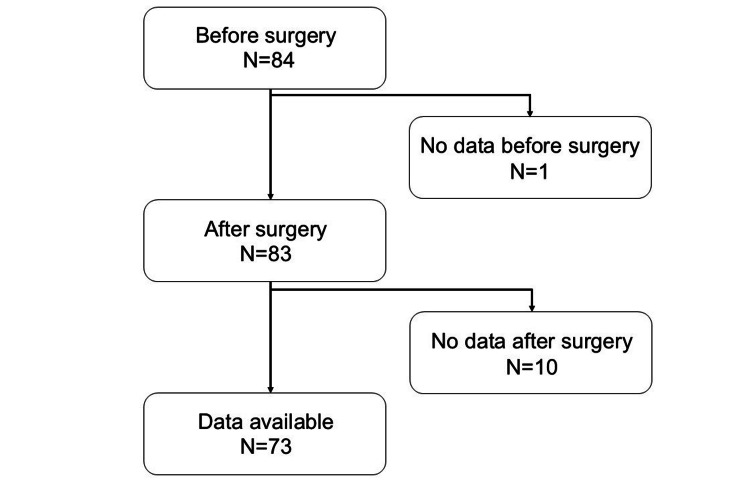
Flowchart of the study

Among 84 patients who had undergone video-assisted lung lobectomy for lung cancer at Shikoku Cancer Center, Matsuyama, Japan, from April 1, 2018, to August 28, 2019, 73 patients, whose clinical measurements before and after the surgery were available, were included in this study (Figure 1). Seventy-three patients in the analysis were able to measure all clinical parameters assessed in the study. Patients with cognitive decline of 20 points or less on the Revised Hasegawa's Dementia Scale (HDS-R) were excluded.

Ethical approval statement

All procedures performed in studies involving human participants were performed following an approved protocol and in accordance with the ethical standards of the Shikoku Cancer Center Ethics Committee (approval no. 2016-129) and the 1964 Helsinki declaration and its later amendments or comparable ethical standards.

Clinical parameters

Clinical parameters such as age, sex, height, presence or absence of COPD, smoking index (Brinkman index), operative time, intraoperative bleeding, duration of postoperative drain placement, lung function, skeletal muscle mass index (SMI), muscle strength, physical activity, and exercise tolerance were analyzed. Measurements were obtained by physical and occupational therapists before surgery and one week after surgery.

Lung function

Percent vital capacity and forced expiratory volume (%) in one second were evaluated using a spirometer (Spiro Shift SP-470, Fukuda Denshi, Tokyo, Japan) with the patients in a seated position. Analyses were conducted before surgery.

Skeletal muscle mass index (SMI)

SMI was measured using a body composition analyzer (InBody S770, InBody Co., Ltd., Seoul, Korea). SMI (kg/m2) was calculated by dividing the limb skeletal muscle mass (kg) by the height squared (m^2^). Measurements were performed before surgery and one week after surgery.

Muscle strength

Muscle strength was measured using handgrip strength. Handgrip strength was measured using a digital dynamometer (TTM, Inc., Tokyo, Japan) with the participant standing upright. The patients had their shoulders adducted and neutrally rotated, elbows fully extended, forearms and wrists in a neutral position, and feet shoulder-width apart. The patients were required to keep the dynamometer away from all parts of the body. Handgrip strength was measured twice on each side, and the average of the maximum values for each side was used. Measurements were obtained before surgery and one week after surgery.

Physical activity

Physical activity was assessed using the International Physical Activity Questionnaire-Short Form (IPAQ-SF) [15]. The data obtained using the IPAQ-SF were converted to metabolic equivalents-minutes per week (METs-min/week), according to the IPAQ scoring protocol. The total sitting time was also measured. Measurements were self-reported by the patients to the IPAQ-SF before surgery.

Exercise tolerance

Exercise tolerance was measured by the 6MWD. The 6MWD was measured according to the method described by the American Thoracic Society [16]. Measurements were performed before surgery and one week after surgery.

Rehabilitation program

Preoperative rehabilitation included lung expansion exercises using incentive spirometry, instructions on the huffing and coughing method necessary for postoperative sputum discharge, aerobic exercise using a bicycle ergometer, and orientation on early postoperative mobilization. The preoperative period was defined as one to three days from the day of admission. Postoperative rehabilitation started on postoperative day 1 and included exercises such as walking in the room or the ward. After the chest tube was removed, aerobic exercise using a bicycle ergometer and stair climbing exercises were started, and the amount of physical activity was gradually increased according to the patient's condition. The ward nurse encouraged the patients to begin walking in the ward from the first day after surgery (this was done at a time other than that spent by the patient in rehabilitation sessions with the physical therapist), and gradually, they increased the walking distance.

Statistical analysis

Patient data were expressed as the sample size required to achieve 80% power at a two-sided level of significance of 5%, with calculations using G*Power 3.1.9.4.

For SMI, muscle strength, and exercise tolerance, comparison of pre- and postoperative findings was performed using paired t-tests and Wilcoxon signed-rank tests. For height, SMI, muscle strength, and exercise tolerance comparisons between males and females were evaluated using Student’s t-test and the Mann-Whitney U test. To assess the factors affecting exercise tolerance after surgery, stepwise multiple regression analyses were conducted using the parameter of postoperative exercise tolerance as an independent variable and those of age, sex, height, COPD, smoking index, operative time, intraoperative bleeding, duration of postoperative drain placement, lung function (before surgery), SMI (before and after surgery), muscle strength (before and after surgery), and physical activity (before surgery) as dependent variables.

IBM SPSS Statistics for Windows, Version 22.0 (released 2013, IBM Corp., Armonk, NY) was used to analyze the data, and differences were considered significant at p < 0.05.

## Results

Clinical characteristics

The patient characteristics are shown in Table 1. The mean age was 68.1 years, gender was 35 males and 38 females, and the mean BMI was 22.8 kg/m^2^.

**Table 1:** Characteristics of the patients a) mean ± standard deviation, b) proportion COPD, chronic obstructive pulmonary disease; %VC, percent vital capacity; FEV1%, forced expiratory volume ％ in one second

Variable	
Age (y) ^a)^	68.1 ± 9.3
Sex (male/ female)^ b)^	35/38
Body mass index(kg/m^2^) ^a)^	22.8 ± 2.8
COPD (yes/no) ^b)^	19/54
Operative time (minute) ^a)^	205.5 ± 63.5
Brinkman Index ^a)^	463.3 ± 551.9
Intraoperative bleeding (ml) ^a)^	88.0 ± 79.7
Duration of postoperative drain placement(day) ^a)^	3.9 ± 2.7
Physical activity (MET- minute /week) ^a)^	1377.9 ± 2057.9
Sitting time (minute /day) ^a)^	356.9 ± 191.7
%VC (%) ^a)^	105.1 ± 14.9
FEV_1_％(%) ^a)^	71.7 ± 8.1

Table 2 shows the preoperative and postoperative changes in SMI, handgrip strength, and 6MWD. No significant changes were observed in SMI before surgery and one week after surgery. However, handgrip strength and 6MWD were significantly lower after surgery than those observed preoperatively (p < 0.05).

**Table 2:** Comparisons of physical function and six-minute walk distance before and after surgery for lung cancer mean ± standard deviation SMI, skeletal muscle mass index; 6MWD, six-minute walk distance

Item	Preoperative	Postoperative	p-value
SMI (kg/m^2^)	6.6 ± 1.0	6.5 ± 1.0	0.208
Handgrip strength (kg)	26.9 ± 8.6	25.8 ± 8.7	p < 0.0001
6MWD (m)	474.0 ± 87.2	444.3 ± 85.8	p < 0.0001

Table 3 shows differences in height, grip strength, and SMI between males and females. Males were significantly taller than females (p < 0.05). Preoperative and postoperative grip strength and SMI were significantly higher in males than in females (p < 0.05).

**Table 3:** Differences in height, grip strength, skeletal muscle mass index, and six-minute walk distance between males and females mean ± standard deviation SMI, skeletal muscle mass index; 6MWD, six-minute walk distance

Item	Male	Female	p-value
Height (cm)	165.3 ± 5.0	154.0 ± 5.2	p < 0.0001
Preoperative handgrip strength (kg)	33.0 ± 7.9	21.4 ± 4.4	p < 0.0001
Postoperative handgrip strength (kg)	32.2 ± 7.8	19.9 ± 4.2	p < 0.0001
Preoperative SMI (kg/m^2^)	7.3 ± 0.6	5.8 ± 0.6	p < 0.0001
Postoperative SMI (kg/m^2^)	7.3 ± 0.6	5.8 ± 0.6	p < 0.0001
Preoperative 6MWD (m)	486.2 ± 78.1	462.7 ± 94.4	0.253
Postoperative 6MWD (m)	455.7 ± 82.9	433.7 ± 88.2	0.276

Factors affecting postoperative exercise tolerance

Table 4 shows the results of the multiple regression analyses. Stepwise multiple regression analyses showed that higher postoperative handgrip strength, males, and higher preoperative physical activity had a significant positive effect on postoperative 6MWD (p < 0.05, R^2^ = 0.348).

**Table 4:** Factors predicting the six-minute walk distance at one week after surgery 6MWD, six-minute walk distance; †, variables were selected by backward stepwise multiple regression models; β, standardized regression coefficient; CI, confidence interval

Item	Included variable †	β	95% CI	*Adjusted* R^2^	p-value
6MWD				0.348	
	Handgrip strength one week after surgery	0.715	4.331-9.745		p < 0.00001
	Sex	0.388	112.530-19.917		0.006
	Physical activity	0.243	0.002-0.018		0.015

## Discussion

In this study, we aimed to identify the factors related to walking endurance in patients who underwent VATS for lung cancer. Few studies have been conducted to analyze the factors associated with the 6MWD in patients who underwent VATS for lung cancer. Our results showed that handgrip strength after surgery, sex, and physical activity before surgery were associated with exercise tolerance.

Moriello et al. reported that the 6MWD reduced, on average, from 478 m preoperatively to 429 m three weeks postoperatively in patients undergoing gastrointestinal surgery [17], and in patients who underwent surgery for lung cancer, the 6MWD has been shown to decrease significantly within 14 days of surgery [5]. Postoperative maximal oxygen uptake (VO2 max) reduced to 28 % (a 19 % decrease) during the 14 days after surgery [18,19]. Exercise tolerance decreases further within six months of the lung cancer treatment; this decrease is observed subsequent to a decline in lower-extremity muscle function, reduction in physical activity, and worsening symptoms [20]. Clinical improvements in the 6MWD have been shown to range from 30 to 54 m [21,22]. In this study, the 6MWD significantly decreased from 474 m before surgery to 444 m one week after surgery. Furthermore, handgrip strength significantly decreased one week after surgery compared to that before surgery. Patients with lung cancer who underwent VATS also required home-based exercise and outpatient rehabilitation interventions after discharge because of decreased endurance and muscle strength at discharge. However, the present study was conducted over a short period of time, and a longer-term, ongoing study after returning to home should be conducted.

Patients with low preoperative physical activity levels showed slow progression to postoperative mobilization, which is detrimental to the improvement in physical function postoperatively [23]. In our study, physical activity levels before surgery strongly influenced the 6MWD. Moderate- to vigorous-intensity physical activity is significantly and positively associated with physical function, including gait speed and balance ability, whereas sedentary behavior is significantly and negatively associated with physical function [24]. Moreover, physical activity had a positive effect on the postoperative 6MWD, as it caused an increase in the patient's physical function and exercise tolerance. Preoperative exercise therapy has a beneficial effect because it enhances post-surgery recovery [25]. Effective interventional strategies are needed to implement preoperative rehabilitation programs for cancer patients because of the limited time available between diagnosis and surgery. For preoperative rehabilitation intervention in patients scheduled for surgery for lung cancer, we need to focus not only on improving functional capacity but also on increasing their physical activity levels to prevent a postoperative decline in the 6MWD. Our findings also demonstrated that assessment of preoperative physical activity may be useful in predicting the 6MWD in patients with lung cancer. Moreover, assessment of preoperative activity levels will help in the assessment of the need for preoperative physical therapy to prevent a decrease in the 6MWD after surgery. In this study, the IPAQ was used to evaluate physical activity, but a pedometer can be used for a more detailed evaluation. The subject's use of a pedometer for evaluation can also self-confirm the amount of physical activity. In the future, it is also necessary to consider intervention methods to increase the amount of physical activity for patients.

Lower-extremity muscle function is a determinant of exercise tolerance after lung resection surgery in patients with lung cancer [26]. Handgrip strength is positively associated with overall body strength [27]. In our study, postoperative handgrip strength was observed to be a factor influencing the 6MWD. Postoperatively, the 6MWD is affected by muscle strength, including lower-extremity muscle strength, and rehabilitation strategies such as early mobilization and muscle-strengthening exercises are necessary.

Males walked greater 6MWD than females [28], and males also have greater muscle strength and muscle mass than females [29]. The results of this study supported previous studies, as males had a positive effect on the 6MWD, and males had significantly higher height, muscle strength, and muscle mass than females.

Finally, there were some limitations to the present study. First, it is necessary to examine not only the week after surgery but also the long-term impact after discharge. Second, the patients in this study did not undergo an in-hospital assessment of physical activity, such as the use of pedometers for analyses. Third, since the study was conducted at a single institution, patient conditions and intervention methods may have been influenced by the characteristics of the institution, and therefore, a multicenter intervention is required. Fourth, we have not been able to examine factors affecting exercise tolerance by gender because of the small number of patients. Fifth, there are other factors that affect the 6MWD other than those we studied, and further investigation is needed to include other items as well. Further research is needed to examine these factors.

## Conclusions

Improving postoperative exercise tolerance is also one of the goals of rehabilitation. The intervention to increase the amount of preoperative physical activity is needed because postoperative exercise tolerance was affected by the amount of preoperative physical activity. In addition, postoperative interventions that focus on muscle strength are required while also implementing mobilization because muscle strength also affects postoperative exercise tolerance.
